# Invasion of a Shared Space: The Nightmare Roommate

**DOI:** 10.1016/j.atssr.2022.11.001

**Published:** 2022-11-08

**Authors:** Stephanie N. Nguyen, Isao A. Anzai, Nicholas J. Shea, Andrea Miltiades, Jay Leb, Hiroo Takayama

**Affiliations:** 1 Division of Cardiac, Thoracic, and Vascular Surgery, NewYork-Presbyterian/Columbia University Irving Medical Center, New York, New York; 2 Department of Anesthesiology, NewYork-Presbyterian/Columbia University Irving Medical Center, New York, New York; 3 Department of Radiology, NewYork-Presbyterian/Columbia University Irving Medical Center, New York, New York

## Abstract

Shared sheath rupture is a rare manifestation of acute aortic syndrome. An aortic rupture into the common subadventitial space of the great vessels leads to hematoma extension along the pulmonary arterial tree, thus mimicking a pulmonary embolism. We report a case of shared sheath rupture promptly diagnosed on cross-sectional imaging and managed successfully with a root and hemiarch replacement.

Because the great vessels share a common adventitial layer beginning at the level of the root, rupture of the proximal aorta may lead to hemorrhagic extravasation into the shared sheath and along the pulmonary arterial tree. The resultant pulmonary artery (PA) intramural hematoma may extend across the bifurcation, causing extraluminal compression of the central PAs. On radiographic examination, shared sheath rupture may be misinterpreted as a pulmonary embolism (PE) because of luminal narrowing, which can have serious repercussions from unnecessary anticoagulation or thrombolysis. Therefore, when shared sheath rupture is suspected, careful assessment for aortic root disease must be undertaken. We present a case of contained aortic rupture into the aortopulmonary subadventitial space, mimicking a PE on imaging.

A 67-year-old man presented with 10 days of worsening chest pain. Computed tomography angiography demonstrated a 7.2-cm ascending aorta with a focal tear and rupture along the left lateral aspect of the mid-ascending aorta (Figure 1). The nonopacified false lumen extended proximally to the aortic root and distally into the distal arch. There was evidence of rupture into the shared aortopulmonary adventitial sheath with blood tracking along the central PAs, resulting in moderate narrowing of the main PA and severe narrowing of the right PA. At first glance, there appeared to be a saddle PE; however, careful review of the imaging confirmed extraluminal compression of the PA secondary to extravasated blood from an aortic rupture (Figure 2). The patient remained hemodynamically stable and was prescribed anti-impulse therapy before undergoing emergent hemiarch and root replacement.

**Figure 1:**
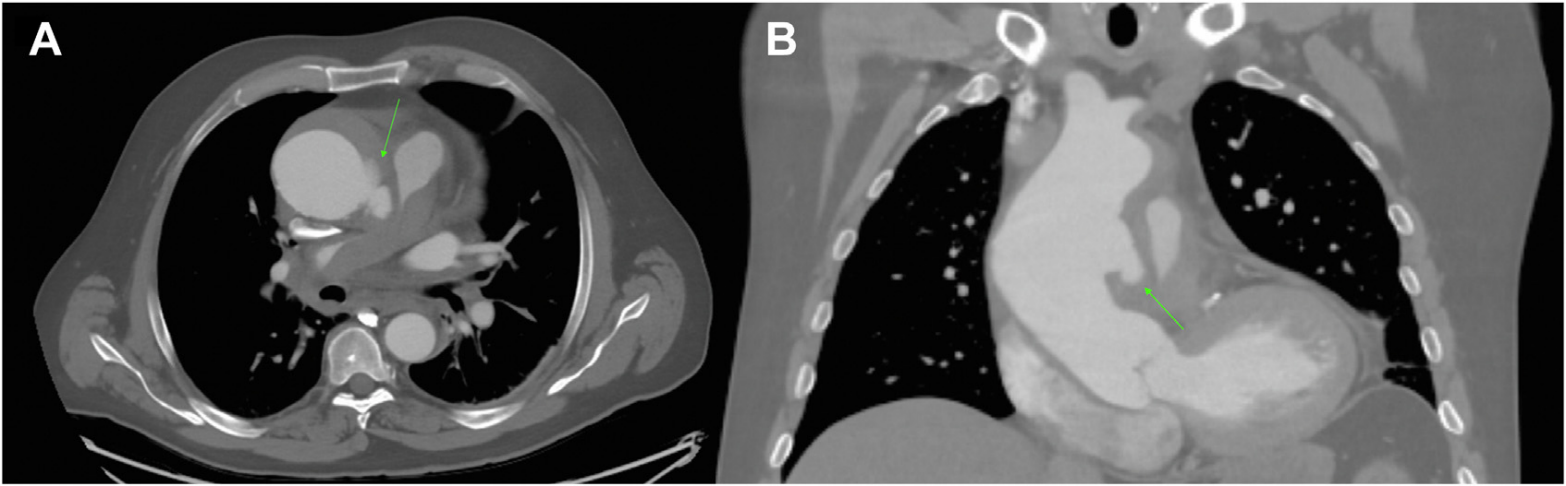
(A) Axial and coronal (B) contrast-enhanced computed tomography image demonstrating a focal aortic tear and rupture along the left lateral aspect of the mid-ascending aorta (green arrows) into the shared adventitial sheath of the great vessels.

**Figure 2:**
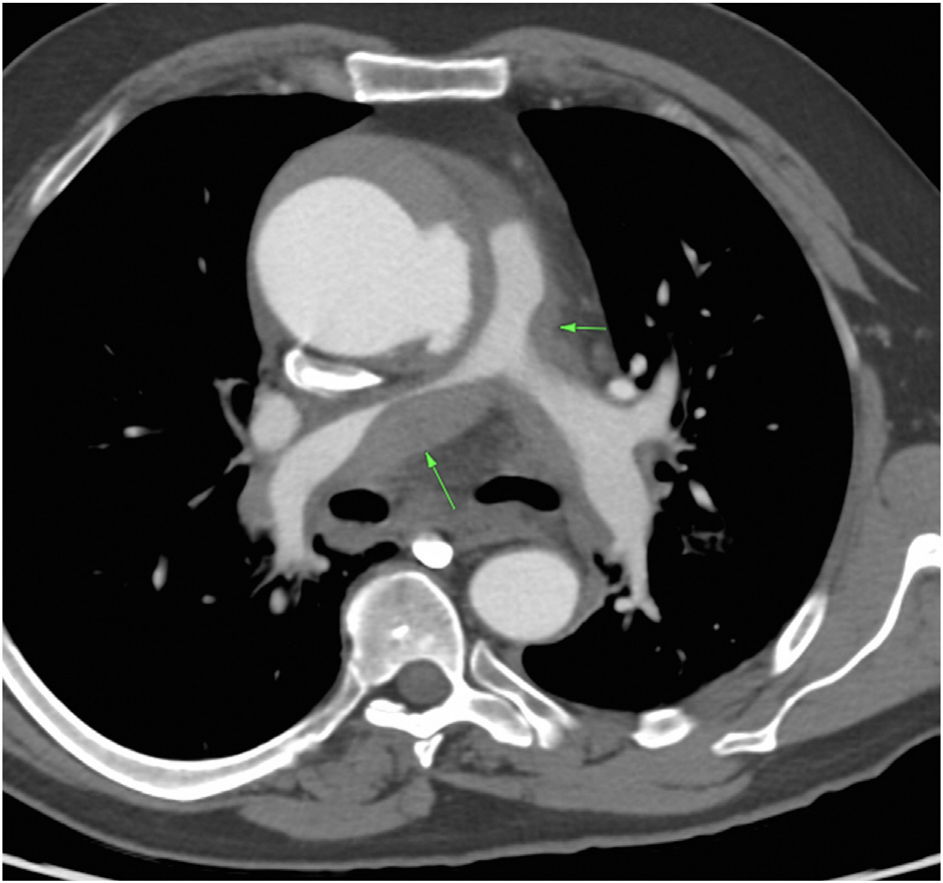
Axial contrast-enhanced computed tomography image demonstrating a large volume of high-attenuation material tracking along the central pulmonary arteries, consistent with intramural hematoma. There is moderate narrowing of the main pulmonary artery and severe narrowing of the mid right pulmonary artery (green arrows). This may be easily misinterpreted as a saddle pulmonary embolism if proximal aortic disease is missed.

Intraoperative transesophageal echocardiography revealed severe left ventricular dysfunction, preserved right ventricular function, moderate aortic insufficiency, and significant narrowing of the right PA. A median sternotomy was performed and the patient was cooled to 24 °C. The hemopericardium was slowly decompressed, revealing a massively dilated aorta with purple discoloration. The ascending aorta was cannulated under epiaortic ultrasound guidance by Seldinger technique, followed by bicaval venous cannulation and placement of a retrograde cardioplegia catheter and left ventricular vent. The circulation was arrested, and retrograde cerebral perfusion was initiated. The arterial cannula was removed, and the aorta was transected at the level of the innominate artery takeoff and beveled beneath the arch vessels. Direct ostial and retrograde del Nido cardioplegia was administered. Unilateral antegrade cerebral perfusion was initiated through the innominate artery.

A transverse intimal tear was noted in the mid-ascending aorta with a dissection flap extending from the anterior root to the proximal arch; fresh thrombus filled the false lumen. The tear was completely excised. Posteriorly, there was a large hematoma tracking along the aortic and pulmonic walls, completely obscuring the adventitial layer and suggestive of a contained rupture. A plane was carefully developed along the lesser curvature of the arch. The proximal transverse arch was anastomosed to a 28-mm Gelweave 1-branched graft (Vascutek) using a double layer of 4-0 Prolene with felt strip reinforcement. Bypass was resumed through the graft sidearm, and full rewarming commenced.

Inspection of the root was notable for a prominent hematoma involving the left and right sinuses and PA wall. The integrity of the aortic adventitia was violated in multiple areas in the aortopulmonary window, confirming the rupture and dissection into the PA wall. It was clear that a root replacement was necessary. For the nonviolated adventitia to be preserved, gentle handling of the friable sinus tissue was critical as sinus remnants of each coronary button contained hematoma. Notably for the left sinus, the dissection plane was developed along the wall of the PA, leaving all the soft tissue with the left coronary button. Because the presence of a high-profile, rigid valve prosthesis in a prefabricated conduit may limit visualization in this challenging coronary reconstruction, we performed an in situ composition of a valved conduit, a technique previously described by our group to manage complex root replacements.^1^ A 34-mm Valsalva graft (Terumo Aortic) was inverted and inserted into the left ventricular outflow tract. The graft was sewn circumferentially to the left ventricular outflow tract, then retrieved outward. The large coronary buttons were reimplanted with large bites of 4-0 Prolene, incorporating the hematoma and adventitia, reinforced with autologous pericardial strips. A No. 25 Inspiris valve (Edwards Lifesciences) was then seated within the graft. After completion of the graft-to-graft anastomosis, the cross-clamp was removed and the patient weaned off bypass uneventfully.

## Comment

Shared sheath rupture is a rare complication of acute aortic syndrome due to a contained posterior root rupture with tracking of blood along the PA tree through a common adventitial layer. This results in extraluminal compression because the intrapulmonary artery pressure is low. Prompt recognition of this complication is necessary as severe obstruction can lead to acute pulmonary hypertension and right-sided heart failure, which when taken together with the radiographic findings may lead to inappropriate anticoagulation or thrombolytic therapy for PE.^2^

A thorough review of computed tomography imaging is essential, particularly when a patient is diagnosed with a simultaneous acute aortic syndrome and PE or when the aortic intimal flap is not visualized. In such situations, attention should be directed to the aortic root to identify a site of rupture (usually posterior) into the shared sheath. A direct communication between the aorta and PA with active extravasation of contrast material is rare, and more commonly, there will be high-attenuating hematoma tracking eccentrically along the wall of the central PA.^2^ From here, blood can continue to dissect out to the peripheral PAs, potentially causing alveolar or parenchymal hemorrhage.^3^ The long-term sequelae of shared sheath rupture on the pulmonary vasculature are unknown, with some reports of pulmonary hypertension and aneurysmal PA dilation secondary to elevated wall stress from chronic intramural hematoma.^4^

From a surgical standpoint, aortic tissue integrity is exceedingly low in cases of shared sheath rupture, more so than in a standard aortic dissection. There is near-total obliteration of the adventitial layer, which makes the harvest and reimplantation of coronary buttons technically challenging—these steps must be perfect. As mentioned, the presence of a high-profile, rigid valve prosthesis in a prefabricated conduit may hinder visualization. Our technique of in situ composition of a valved conduit^1^ was extremely helpful in this case; in addition to maximizing visibility during coronary button reimplantation, this technique enables the surgeon to freely maneuver the graft to facilitate the anastomosis while minimizing manipulation of the fragile button.

Overall, shared sheath rupture is a rare manifestation of acute aortic syndrome; however, the radiologic findings are characteristic. Understanding that the great vessels are “roommates” within a shared adventitia and prompt recognition of a posterior aortic rupture while excluding PE are paramount.
